# When Paying for Care Becomes a Crisis: Affordability Barriers, Coping Strategies, and Financial Consequences of Chronic Illness in Lagos, Nigeria

**DOI:** 10.7759/cureus.112226

**Published:** 2026-07-07

**Authors:** Chineye F Adili-George, Uchechi N Obiefule, Ruth O Adili-George, Uwemedimo S Isaiah, Uchenna A Amaechi

**Affiliations:** 1 Health Professions, Health Services Administration, University of Alabama at Birmingham, Birmingham, USA; 2 Nursing, Lagos State University Teaching Hospital, Lagos, NGA; 3 International Management, Rome Business School Nigeria, Lagos, NGA; 4 Psychology, University of Uyo, Uyo, NGA; 5 Global Health, University of Geneva, Geneva, CHE

**Keywords:** affordability barriers, distress financing, nigeria, non-communicable diseases, out-of-pocket payments, universal health coverage

## Abstract

Background: Chronic non-communicable disease (NCD) care in Nigeria remains largely financed out of pocket, exposing households to affordability barriers and harmful coping strategies. This study examined the prevalence and patterning of affordability barriers, financial coping strategies, and predictors of foregone care and distress financing among chronic NCD patients attending public health facilities in Lagos, Nigeria.

Methods: A facility-based cross-sectional survey was conducted among 480 adults receiving outpatient care for chronic NCDs, such as hypertension, type 2 diabetes, cardiovascular disease, chronic kidney disease, cancer, chronic respiratory conditions, and sickle cell disease across primary, secondary, and tertiary public facilities. Participants were recruited using a multistage sampling approach, with eligible patients selected systematically from clinic registers. Data were collected using a structured, interviewer-administered questionnaire capturing sociodemographic and household characteristics, employment status, chronic NCD diagnosis and treatment duration, out-of-pocket healthcare expenditure, affordability barriers, and financial coping strategies. Affordability outcomes included being offered unaffordable treatment, cost-driven treatment decisions, irregular clinic attendance, and inability to access care because of inability to pay. Financial coping strategies were grouped as self-directed financing, social-network support, and distress financing, defined as borrowing or the sale of assets. Prevalence estimates were reported with exact 95% CIs. Facility-tier differences and subgroup associations were assessed using chi-square, Fisher's exact, and ANOVA tests. Binary logistic regression models identified predictors of foregone care and distress financing, with average marginal effects and adjusted predicted probabilities estimated to aid interpretation. Statistical significance was set at a two-sided p<0.05.

Results: Affordability barriers were common: 42.9% of patients had been offered unaffordable treatment, 41.9% reported that cost shaped treatment decisions, and 45.0% had forgone care because they could not pay. Among affordability challenges, medication unaffordability was especially prominent and was comparable across facility tiers, suggesting system-wide pharmaceutical cost pressures rather than isolated access gaps. Coping followed a clear financial hierarchy: patients first relied on savings or household income (82.9%), many then mobilized social-network support (69.6%), and a substantial minority resorted to distress financing through borrowing or asset sale (28.5%). Financial-exposure intensity and treatment duration independently predicted distress financing; financial-exposure intensity also predicted foregone care. Pension income reduced the probability of distress financing by 15.2 pp, while insurance showed no detectable protection.

Conclusion: Chronic NCD care in Lagos public facilities becomes a financial crisis through pervasive affordability barriers, prominent medication unaffordability, reliance on informal household coping, financial-exposure intensity, and treatment chronicity, which drive care rationing and distress financing. Nigeria's universal health coverage agenda should include scaling insurance enrolment for chronic NCD patients, benefit-package redesign, pharmaceutical-pricing reform, and income-protection mechanisms that absorb the recurrent costs of chronic illness care.

## Introduction

Chronic non-communicable diseases (NCDs), including cardiovascular disease, cancer, diabetes, and chronic respiratory conditions, now account for 74% of deaths globally, with 82% of premature NCD deaths occurring in low- and middle-income countries (LMICs) [1]. Sub-Saharan Africa is experiencing one of the fastest NCD transitions, with chronic disease rising rapidly while many health systems remain organized around episodic rather than long-term care [2,3]. Nigeria, the region's most populous country and one of its most under-insured health systems, illustrates the financial-protection gap created by this transition. Out-of-pocket payments still account for more than 70% of health spending, federal health allocations have remained close to 4% of public expenditure, and about 30% of households affected by NCDs experience catastrophic health expenditure (CHE) when measured against the WHO 40%-of-non-food-expenditure threshold [4,5], one of two commonly used CHE metrics in the financial-protection literature.

Affordability barriers are often the first point at which this burden becomes visible. Affordability has been conceptualized as the fit between the cost of seeking care and a household's capacity to pay; when that fit fails, care is delayed, modified, or abandoned [6]. Nigerian evidence suggests that this failure is common. More than two-thirds of diabetes patients in a tertiary setting have reported care as unaffordable [7]; older adults using Lagos primary care centers identify laboratory and medication costs as major barriers [8]; and among hypertensive patients in Ekiti, medication adherence varies inversely with the cost of admissions and admission-related medicines [9]. A West African scoping review similarly identifies high care costs and supply gaps as major barriers to diabetes primary care across the sub-region [10]. However, this literature has not shown how affordability barriers differ by facility tier, financial exposure, and disease complexity within a single Nigerian chronic-NCD cohort.

When cost does not prevent care altogether, households must find ways to absorb it. The McIntyre framework describes a progression from formal and self-directed coping, such as savings, household income, and insurance, through social-network support from relatives, friends, and religious institutions, to distress financing through borrowing, asset sales, and other debt-generating strategies [11]. Evidence from South Asia shows distress financing as a frequent response to NCD costs, with reported prevalence ranging from 58% in Bangladesh hospitalization studies [12] to 72.4% in Indian breast cancer care [13], while Tanzanian informal-sector evidence shows heavy reliance on cash, borrowing, and asset sales among populations outside insurance protection [14]. In sub-Saharan Africa, however, evidence on coping strategies remains limited. A recent scoping review found that NCD coping data are concentrated in a small number of countries and are often measured using broad composite indicators that obscure differences by tier, subgroup, and coping pathway [15]. In Nigeria, the pattern of distress financing among chronic NCD outpatients remains poorly characterized.

The question of which patients forgo care and which incur debt has largely been studied through national household surveys. These studies identify rural residence, low socioeconomic status, lack of insurance, larger household size, and chronic illness as population-level predictors of catastrophic expenditure [16]. At the facility level, the question is different: after accounting for patient characteristics, do enabling resources such as insurance, pension income, and employment protect against foregone care and distress financing, or is financial exposure itself the dominant driver of hardship? Andersen's behavioral model of healthcare use provides the access framework for this question by distinguishing predisposing characteristics, enabling resources, and need factors [17]. Its application to Nigerian chronic NCD outpatients, particularly in relation to whether insurance and pension income reduce point-of-care hardship, remains limited.

The Nigerian evidence base therefore documents hardship in broad terms but offers less clarity on the mechanisms that produce it at the patient-facility interface. Existing studies rarely show whether protective instruments such as insurance, pension income, and formal employment reduce hardship in chronic NCD care, or whether their protective effect is weakened because recurrent medication and diagnostic costs remain outside the effective coverage envelope. It also remains unclear whether financial-exposure intensity, rather than demographic vulnerability, is the more immediate driver of both foregone care and distress financing, and whether hardship varies by facility tier, disease complexity, or financial-exposure subgroup. These questions are especially important as the 2022 National Health Insurance Authority (NHIA) Act is being implemented in a context where evidence on chronic NCD financial protection remains incomplete [18,19].

This study addresses this gap by drawing on Andersen's behavioral model [17] and the McIntyre-Cleary financial-coping framework [6,11] to examine affordability barriers, coping strategies, and predictors of foregone care and distress financing among 480 chronic NCD patients attending primary, secondary, and tertiary public facilities in Lagos, Nigeria. Specifically, it asks the following: First, what is the prevalence and nature of affordability barriers, and how do these vary by facility tier, financial exposure, and disease complexity? Second, what coping strategies do patients use to meet chronic illness costs, and how common is distress financing across subgroups? Third, which sociodemographic and financial factors independently predict foregone care due to cost and distress financing?

## Materials and methods

Study design and setting

A facility-based cross-sectional survey across the primary, secondary, and tertiary tiers of the public health system of Lagos State, the most populous and economically dynamic state in Nigeria. The state houses an estimated resident population of over 22 million [20] distributed across 20 local government areas (LGAs) organized into three senatorial districts - Lagos Central, Lagos East, and Lagos West [21]. Lagos operates a mixed public-private health system structured into the standard three-tier configuration, and the Nigeria Health Facility Registry lists more than 2,207 facilities in the state, 81% of which fall under public-sector administration [22]. Reporting of this study follows the Strengthening the Reporting of Observational Studies in Epidemiology (STROBE) guideline for cross-sectional observational studies.

Study population and eligibility criteria

The study enrolled adults aged 18 years or older with a clinically confirmed diagnosis of one or more chronic NCDs, including hypertension, type 2 diabetes, cardiovascular disease, chronic kidney disease, cancer, chronic respiratory conditions, bone or joint disease, chronic liver disease, mental health conditions and sickle cell diseases who had been receiving ongoing outpatient care at the participating facility for a minimum of three months. Patients presenting for acute illness, those clinically unstable for survey interview, and those lacking a confirmed chronic diagnosis meeting the duration threshold were ineligible.

Sample size determination

We derived the minimum sample size from the standard single-population proportion formula [23]:

$n = \frac{Z^2 \cdot P \cdot (1 - P)}{E^2}$

Here, Z = 1.96; p = 0.5 (the most conservative prevalence assumption in the absence of tier-stratified affordability-barrier estimates); and E = 0.05; this returned 384 participants. We inflated the target by 20% to absorb anticipated non-response, arriving at 480 patients.

Sampling strategy

Facility and participant selection followed a five-step multistage sampling technique. We stratified Lagos State by senatorial district [21], drew two LGAs from each district through computer-generated random selection (six LGAs total), and purposively selected within each LGA one public facility securing tier representation. The realized mix comprised two primary, three secondary, and one tertiary facility, with the 480-participant total allocated equally across the six facilities (80 each; 160 primary, 240 secondary, 80 tertiary). Within each facility, we recruited eligible patients attending the General or Medical Outpatient Department through systematic random sampling against the daily clinic attendance register, deriving the sampling interval as the ratio of eligible attendees to the facility quota.

Data collection

Fieldwork for this study ran from December 2024 through February 2025. We deployed a structured, interviewer-administered questionnaire adapted from the validated instrument used by Okediji et al. in their facility-based assessment of the economic impacts of chronic illness on households in south-western Nigeria [24], with item construction additionally informed by the affordability framework of Cleary et al. [6] and the household financial-coping framework of McIntyre et al. [11]. The questionnaire captured sociodemographic and household characteristics, employment, chronic NCD diagnosis and treatment duration, out-of-pocket healthcare expenditure, affordability barriers, and financial coping strategies. The study instrument was administered in English, Yoruba, Igbo, or Nigerian Pidgin, according to participant preference. Six trained research assistants administered the questionnaire face-to-face after a four-day training program; pilot-tested the instrument at a non-study public facility prior to main fielding; and two field supervisors conducted a same-day data-quality review.

Variable operationalization

Affordability was assessed using four indicators: being offered treatment that the respondent could not afford; the main driver of treatment decisions, categorized as cost, clinical guidance, or family influence; clinic non-attendance and its stated reason; and inability to obtain care because of inability to pay. The latter served as the primary care-access outcome and was coded as present for responses of "sometimes," "often," or "almost always," and absent for "seldom," "never," or "pays extra and manages."

Financial coping was measured using a multiple-response item on sources used to pay for care. Guided by the McIntyre-Cleary framework [6,11], responses were grouped into formal or self-directed coping, social-network coping, and distress financing. The primary distress-financing outcome combined borrowing for treatment and the sale of assets or property. A coping-diversity score, ranging from 0 to 11, captured the number of financing strategies used.

Predictors were selected using Andersen's behavioral model [17]. Predisposing factors included age, sex, and education; enabling factors included employment, insurance status, pension status, household size, and facility tier; and need factors included multimorbidity and treatment duration. Financial-exposure intensity was modeled as log-transformed total out-of-pocket expenditure (OOPE), log(OOPE+1).

Subgroup variables included CHE, impoverishment, multimorbidity, and insurance status. CHE was operationalized using the World Bank financial-protection metric, defined as out-of-pocket healthcare expenditure equal to or exceeding 10% of total household income [25]. This differs from the WHO capacity-to-pay approach earlier cited in the Introduction [5], which defines catastrophic expenditure as health spending exceeding 40% of non-food expenditure. We selected the 10%-of-household-income threshold because it aligns with the household-income data collected in this study and is widely used for financial-protection monitoring [25]. Impoverishment was defined as post-payment per-capita income below the World Bank 2022 international poverty line of US$2.15 per person per day [26], equivalent to NGN 3,440 per day using an approximate study-period exchange rate of NGN 1,600/US$. Multimorbidity was defined as two or more chronic conditions, and insurance status was classified according to whether participants reported any health insurance coverage. To reduce recall bias and improve the accuracy of self-reported expenditure data, respondents were asked to report expenditures with reference to their most recent NCD-related care episodes. Reported figures were cross-checked against receipts, electronic payment statements, pharmacy records, and clinical or facility records where these were available.

Statistical analysis

Analyses were conducted in Stata (version 19; StataCorp LLC, College Station, TX) after de-identification of the analytic dataset. Prevalence estimates were reported with exact binomial 95% confidence intervals using the Clopper-Pearson method. Facility-tier differences were assessed using chi-square tests, with Fisher's exact test applied where expected cell counts were below five, and one-way ANOVA used for continuous variables. Bivariate associations between affordability and coping outcomes and key financial or clinical characteristics, including catastrophic expenditure, impoverishment, multimorbidity, and insurance status, were assessed using cross-tabulations with chi-square or Fisher's exact tests as appropriate.

Binary logistic regression models were fitted on the full sample (n=480), beginning with full nine-predictor specifications and retaining predictors significant at p<0.10 in the full model; parsimonious specifications were confirmed by nested likelihood-ratio tests against the full model (all p>0.25). Treatment duration was retained as a block in the Model B parsimonious specification (joint Wald χ²(3)=6.37, p=0.095; individual categories: 1-4 years p=0.015, 5-9 years p=0.057), consistent with the p<0.10 retention threshold and standard practice of evaluating multi-level predictors as indivisible units rather than selectively retaining individual categories. Events-per-variable ratios were used to assess model stability, while average marginal effects and adjusted predicted probabilities were computed to support interpretation. Because both outcomes exceeded 10% prevalence, modified Poisson regression with robust standard errors was conducted as a sensitivity analysis; substantive conclusions were unchanged. Statistical significance was set at two-sided p<0.05.

Ethical approval

The study protocol received ethical clearance from the Research and Ethics Committee of General Hospital Ikorodu (Reference: GHIKD/REC/35522). Every participant provided written informed consent prior to enrolment. We provided no monetary compensation to avoid coercive enrolment within a financially constrained patient population. All procedures conformed to the principles of the World Medical Association Declaration of Helsinki [27].

## Results

Sample characteristics

Table 1 shows the full sociodemographic, clinical, and financial profiles of the 480 patients that we recruited across the six public facilities. The sample was predominantly female (60.2%) and middle-aged (mean: 56.5 years, SD: 14.9); 69.0% were employed or self-employed, 45.6% held tertiary qualifications, and the average household contained 4.97 members. Formal financial protection was strikingly limited: 85.8% were uninsured, and only 10.4% received pension income, with tertiary-tier patients somewhat more likely to be pensioners (17.5% vs 7.9-10.6% at the lower tiers; p=0.052). Multimorbidity affected 25.4% of patients. Mean OOPE climbed sharply across tiers, from NGN 45,180 at primary to NGN 150,380 at tertiary (p<0.001), and 68.1% of participants crossed the catastrophic-expenditure threshold (54.4% primary, 68.8% secondary, 93.8% tertiary; p<0.001). Post-OOPE impoverishment was, by contrast, uniformly distributed across tiers - 82.5%, 82.9%, and 85.0% (p=0.881) - indicating that financial hardship reached almost the entire cohort, with the tier gradient operating through CHE intensity rather than impoverishment status.

**Table 1:** Sociodemographic, clinical, and financial profiles of chronic NCD patients by healthcare facility tier Chi-square test for categorical variables; one-way ANOVA for continuous variables. CHE1 = OOPE ≥10% of household income; Post-OOPE impoverishment = per capita income below the national poverty line after payment; OOPE = out-of-pocket expenditure; NGN = Nigerian naira. * p<0.05, *** p<0.001.

Variable	Total (n=480)	Primary (n=160)	Secondary (n=240)	Tertiary (n=80)	Test	p
Sex, n (%)						
Female	289 (60.2)	104 (65.0)	146 (60.8)	39 (48.8)	χ²=5.96	0.051
Male	191 (39.8)	56 (35.0)	94 (39.2)	41 (51.2)	-	-
Age, years						
Mean (SD)	56.5 (14.9)	56.4 (13.6)	57.2 (15.0)	54.6 (17.0)	F=0.89	0.411
18-50, n (%)	154 (32.1)	47 (29.4)	73 (30.4)	34 (42.5)	χ²=6.44	0.376
51-65, n (%)	187 (39.0)	70 (43.8)	91 (37.9)	26 (32.5)	-	-
≥66, n (%)	139 (29.0)	43 (26.9)	76 (31.7)	20 (25.0)	-	-
Employment status, n (%)						
Employed or self-employed	331 (69.0)	112 (70.0)	161 (67.1)	58 (72.5)	χ²=0.88	0.644
Not economically active/unemployed	149 (31.0)	48 (30.0)	79 (32.9)	22 (27.5)	-	-
Education level, n (%)						
No formal or primary education	94 (19.6)	32 (20.0)	54 (22.5)	8 (10.0)	χ²=12.23	0.057
Secondary education	167 (34.8)	55 (34.4)	88 (36.7)	24 (30.0)	-	-
Tertiary education	219 (45.6)	73 (45.6)	98 (40.8)	48 (60.0)	-	-
Marital status, n (%)						
Married	297 (61.9)	100 (62.5)	145 (60.4)	52 (65.0)	χ²=4.21	0.649
Widowed, divorced, or single	183 (38.1)	60 (37.5)	95 (39.6)	28 (35.0)	-	-
Household size						
Mean (SD)	4.97 (2.17)	4.93 (2.21)	4.97 (2.16)	5.01 (2.12)	F=0.05	0.951
Health insurance, n (%)						
Uninsured	412 (85.8)	137 (85.6)	210 (87.5)	65 (81.3)	χ²=1.94	0.380
Insured (any scheme)	68 (14.2)	23 (14.4)	30 (12.5)	15 (18.8)	-	-
Pension, n (%)						
No pension income	430 (89.6)	143 (89.4)	221 (92.1)	66 (82.5)	χ²=5.92	0.052
Receiving pension income	50 (10.4)	17 (10.6)	19 (7.9)	14 (17.5)	-	-
Chronic conditions, n (%)						
Single condition	358 (74.6)	125 (78.1)	178 (74.2)	55 (68.8)	χ²=2.52	0.284
Multimorbidity (≥2 conditions)	122 (25.4)	35 (21.9)	62 (25.8)	25 (31.3)	-	-
Treatment duration, n (%)						
Less than 1 year	111 (23.1)	34 (21.3)	48 (20.0)	29 (36.3)	χ²=13.18	0.040*
1-4 years	143 (29.8)	44 (27.5)	74 (30.8)	25 (31.3)	-	-
5-9 years	115 (24.0)	38 (23.8)	63 (26.3)	14 (17.5)	-	-
10 years or more	111 (23.1)	44 (27.5)	55 (22.9)	12 (15.0)	-	-
Financial exposure (OOPE)						
Mean OOPE, NGN (SD)	78,420 (74,310)	45,180 (39,450)	75,640 (68,720)	150,380 (100,640)	F=31.2	<0.001***
Median OOPE, NGN (IQR)	55,000 (28,000-105,000)	32,000 (18,000-65,000)	52,000 (27,000-95,000)	120,000 (75,000-200,000)	-	-
Catastrophic expenditure (OOPE ≥10% income), n (%)	327 (68.1)	87 (54.4)	165 (68.8)	75 (93.8)	χ²=38.17	<0.001***
Impoverishment, n (%)	399 (83.1)	132 (82.5)	199 (82.9)	68 (85.0)	χ²=0.25	0.881
Affordability barriers, n (%)						
Offered treatment could not afford	206 (42.9)	68 (42.5)	104 (43.3)	34 (42.5)	χ²=0.03	0.983
Cost drives treatment decisions	201 (41.9)	80 (50.0)	95 (39.6)	26 (32.5)	χ²=7.75	0.021*
Does not attend clinic regularly	113 (23.5)	57 (35.6)	48 (20.0)	8 (10.0)	χ²=22.80	<0.001***
Ever unable to access care due to cost	216 (45.0)	81 (50.6)	101 (42.1)	34 (42.5)	χ²=3.07	0.215
Financial coping (composite), n (%)						
Own savings or household income	398 (82.9)	136 (85.0)	189 (78.8)	73 (91.3)	χ²=7.50	0.025*
Social network coping (any source)	334 (69.6)	100 (62.5)	175 (72.9)	59 (73.8)	χ²=5.72	0.058
Distress financing (borrowed/sold)	137 (28.5)	47 (29.4)	69 (28.8)	21 (26.3)	χ²=0.27	0.876

Affordability barriers

**Figure 1:**
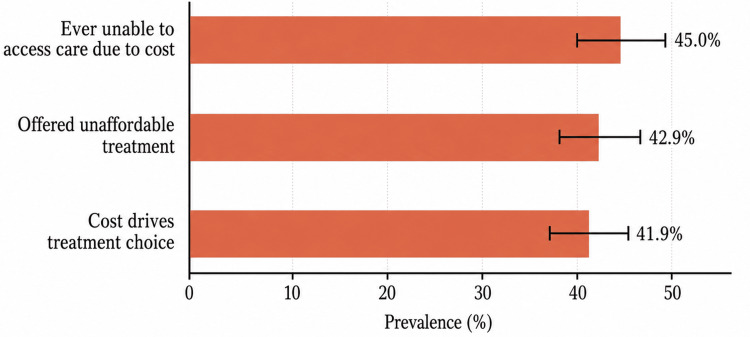
Prevalence of core healthcare affordability barriers among patients with chronic NCDs in public health facilities (N=480) in Lagos, Nigeria All three barriers were significantly associated with catastrophic health expenditure (CHE) status (OOPE ≥ 10% of household income; P < 0.001 for each). Error bars represent 95% Clopper-Pearson confidence intervals.

Table 2 shows the prevalence and tier-distribution of affordability barriers; Figure 1 displays the three headline indicators with confidence intervals. Nearly forty-three percent of patients (42.9%, 95% CI 38.4-47.5%) had been offered treatments they could not afford, with strikingly uniform distribution across tiers (primary 42.5%, secondary 43.3%, tertiary 42.5%; p=0.983). Among the 206 affected patients, drugs and medications dominated as the unaffordable type (57.3%), followed by investigations and diagnostic tests (23.3%), surgery (10.2%), physiotherapy (3.4%), and radiotherapy (1.9%); medication unaffordability showed no significant variation across tiers (54.4% / 60.6% / 52.9%; p=0.621). Cost-driven treatment decisions affected 41.9% of patients with a significant tier gradient declining from 50.0% at primary to 32.5% at tertiary (p=0.021). Nearly a quarter (23.5%) did not attend clinic regularly, again with a marked tier gradient (35.6% primary versus 10.0% tertiary; p<0.001). Forty-five percent (n=216) had foregone care because they could not pay; though the raw rates by tier (50.6%/42.1%/42.5%) did not differ significantly (p=0.215), tier emerged as an independent predictor after adjustment for OOPE in Model A. Among non-attenders (n=113), cost or lack of money was the dominant reason at primary (47.4%) and secondary (52.1%), but fell to 12.5% at tertiary, where "other" reasons (caregiving, distance, drug stock-outs, staff attitudes) dominated.

**Table 2:** Prevalence of affordability barriers to healthcare by facility tier and their association with catastrophic health expenditure and multimorbidity Chi-square test across tiers (p tier); chi-square test of association with catastrophic expenditure status (p CHE) and multimorbidity status (p MM). ‡ Fisher's exact applied due to sparse cells. Types of unaffordable treatment are not mutually exclusive. * p<0.05, *** p<0.001.

Affordability barrier	Total % (95% CI)	Primary %	Secondary %	Tertiary %	p tier	p CHE	p MM
Offered unaffordable treatment, any	42.9 (38.4-47.5)	42.5	43.3	42.5	0.983	<0.001***	0.283
Type of unaffordable treatment (among n=206)							
Drugs or medication	57.3 (50.3-64.0)	54.4	60.6	52.9	0.621	0.621	0.756
Investigations or diagnostic tests	23.3 (17.7-29.7)	26.5	22.1	20.6	0.739	0.150	0.677
Surgery	10.2 (6.5-15.2)	7.4	11.5	11.8	0.639	-	-
Physiotherapy	3.4 (1.4-6.9)	1.5	5.8	0.0	0.228‡	0.228‡	0.228‡
Radiotherapy	1.9 (0.5-4.9)	1.5	1.9	2.9	1.000‡	1.000‡	1.000‡
Treatment decision driver							
Cost or money drives choice	41.9 (37.4-46.4)	50.0	39.6	32.5	0.021*	<0.001***	0.283
Doctor's clinical decision	53.1 (48.5-57.7)	45.0	55.0	63.8	-	-	-
Family decision	5.0 (3.2-7.4)	5.0	5.4	3.8	-	-	-
Clinic attendance							
Does not attend regularly	23.5 (19.8-27.6)	35.6	20.0	10.0	<0.001***	<0.001***	0.208
Reasons for non-attendance (among n=113)							
Lack of money	38.9 (29.8-48.6)	47.4	52.1	12.5	-	-	-
Long distance	15.9 (9.7-24.0)	15.8	18.8	12.5	-	-	-
Forgetting	9.7 (5.0-16.8)	7.0	14.6	0.0	-	-	-
Other reasons	29.2 (21.0-38.5)	21.1	22.9	50.0	-	-	-
Care access due to cost							
Ever unable to access care due to cost, n (%)	45.0 (40.5-49.6)	50.6	42.1	42.5	0.215	<0.001***	0.283
Almost always	3.5 (2.1-5.5)	5.0	2.9	2.5	-	-	-
Often	13.8 (10.7-17.3)	16.3	13.3	10.0	-	-	-
Sometimes	27.7 (23.7-32.0)	29.4	25.8	30.0	-	-	-
Seldom	22.9 (19.2-27.0)	18.1	20.4	40.0	-	-	-
Never	31.7 (27.5-36.1)	30.6	37.1	17.5	-	-	-

Table 3 cross-tabulates these barriers against catastrophic expenditure, impoverishment, multimorbidity, and insurance. The strongest associations are with CHE and impoverishment: unaffordable treatment was reported by 55.1% of CHE patients vs 17.0% of non-CHE (p<0.001), and by 48.9% of the impoverished vs 13.6% of the non-impoverished (p<0.001); cost-driven decisions concentrated similarly (49.5% vs 25.5%, p<0.001; 49.1% vs 6.2%, p<0.001); and inability to access care due to cost reached 55.7% among CHE patients (vs 22.2% non-CHE, p<0.001) and 52.6% among the impoverished (vs 7.4% non-impoverished, p<0.001). Multimorbidity carried a smaller but significant association with offered-unaffordable-treatment (50.8% vs 40.2%, p<0.05), while insurance status showed no protective association with any barrier.

**Table 3:** Affordability barriers among patients with chronic NCDs by catastrophic health expenditure, impoverishment, multimorbidity, and insurance status Chi-square test between subgroup pairs. CHE = OOPE ≥10% of household income. Impov. = post-OOPE per capita income below the national poverty line. MM = multimorbidity (≥2 concurrent chronic conditions). * p<0.05, *** p<0.001.

Affordability barrier	CHE Yes %	CHE No %	Impov. Yes %	Impov. No %	MM Yes %	MM No %	Insured %	Uninsured %
Offered unaffordable treatment	55.1***	17.0***	48.9***	13.6***	50.8*	40.2*	42.6	42.9
Cost drives treatment choice	49.5***	25.5***	49.1***	6.2***	46.7	40.2	38.2	42.2
Does not attend clinic regularly	22.6	25.5	24.6	18.5	20.5	24.6	17.6	24.0
Ever unable to access care due to cost	55.7***	22.2***	52.6***	7.4***	49.2	43.6	48.5	44.7

Financial coping strategies

**Figure 2:**
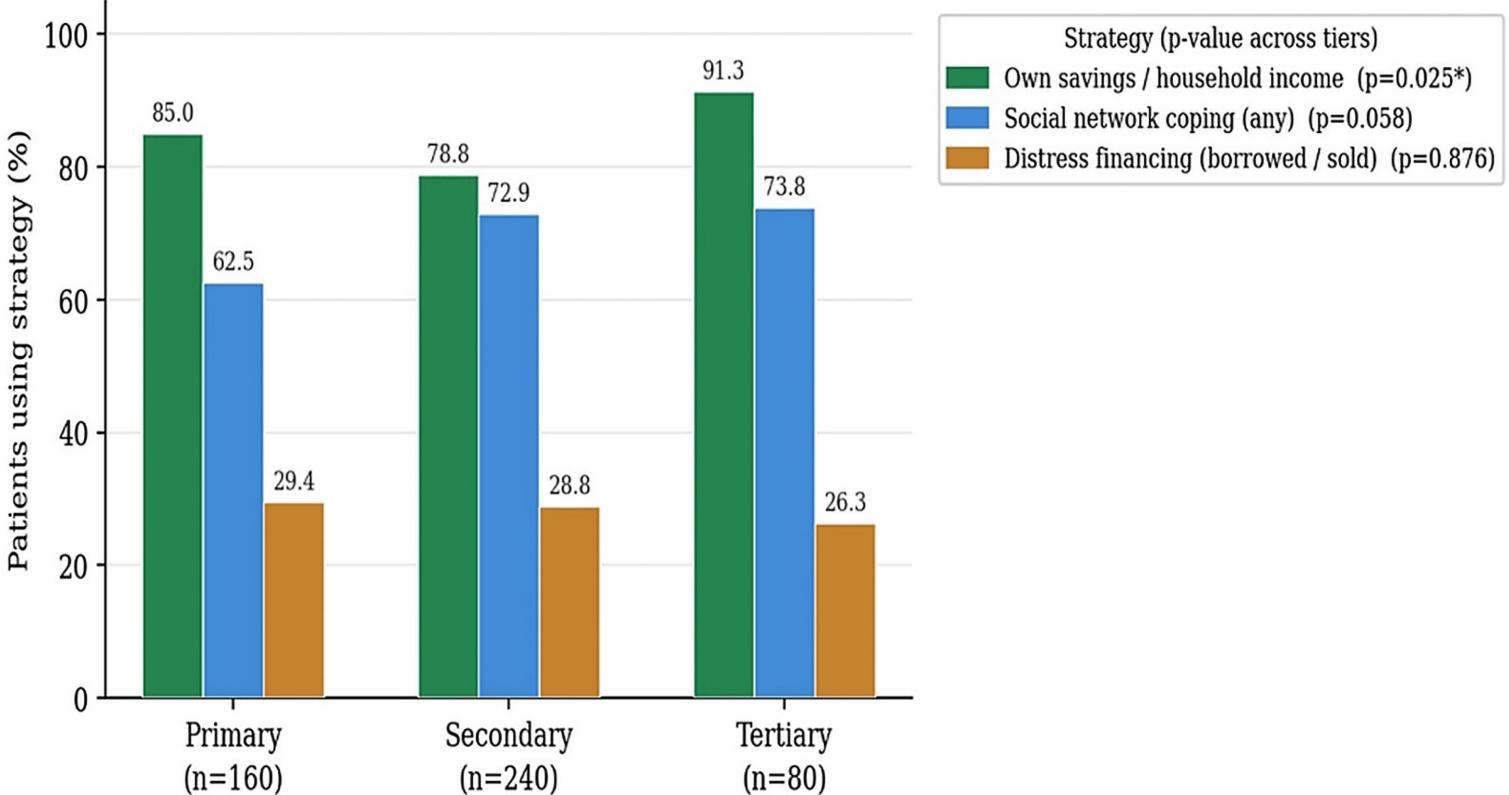
Composite financial coping strategy prevalence by facility tier Lagos public health facilities (N = 480). Three composite strategies are shown: own savings or household income, any social-network coping (relatives, children, friends, employer, church, or mosque), and distress financing (borrowing or the sale of assets).

Table 4 and Figure 2 together describe the financial coping strategies and their tier-distribution. Personal savings or household income was the dominant strategy (82.9%, 95% CI: 79.2-86.2%), with significantly higher reliance at the tertiary level (91.3% vs 85.0% primary and 78.8% secondary; p=0.025). Only 3.8% of the full sample (n=18) actually used their insurance to pay for the current illness, despite 14.2% (n=68) holding any form of insurance - a substantial gap between coverage and uptake. Social network coping from any source reached 69.6% of patients (95% CI: 65.3-73.7%), most commonly contributions from relatives (39.4%) and children (39.0%); within-source tier variation was significant for both - relatives rose from 35.6% at primary to 56.3% at tertiary (p=0.003), while children peaked at secondary (46.7% vs 30.6% primary; p=0.002). Three-quarters of patients used two or more strategies simultaneously, and the average patient drew on 1.99 strategies (SD: 0.92). Distress financing affected 28.5% (95% CI: 24.5-32.7%), of which borrowing was the dominant mode (26.5% borrowed for treatment, 2.5% sold assets). Formal loans from banks or cooperatives - taken specifically to pay for treatment - showed a significant tier gradient declining from 13.1% at primary to 2.5% at tertiary (p=0.019), suggesting primary-tier patients turn most readily to institutional credit when household resources prove insufficient.

**Table 4:** Prevalence of financial coping strategies by facility tier Chi-square test across tiers. Multiple coping strategies could be selected simultaneously. Distress financing composite (Model B outcome) combines borrowing and asset sale. * p<0.05, ** p<0.01.

Coping strategy	Total % (95% CI)	Primary %	Secondary %	Tertiary %	p
Formal and self-directed coping					
Own savings or household income	82.9 (79.2-86.2)	85.0	78.8	91.3	0.025*
Health insurance (used for this illness)	3.8 (2.3-5.9)	2.5	4.2	5.0	-
Social network coping					
Social network coping (any source)	69.6 (65.3-73.7)	62.5	72.9	73.8	0.058
Contributions from relatives	39.4 (35.0-43.9)	35.6	36.3	56.3	0.003**
Contributions from children	39.0 (34.6-43.5)	30.6	46.7	32.5	0.002**
Contributions from friends	15.0 (11.9-18.5)	15.0	14.2	17.5	0.770
Contributions from church	4.8 (3.1-7.1)	5.0	4.6	5.0	0.977
Contributions from mosque	1.0 (0.3-2.4)	1.3	0.8	1.3	0.904
Contributions from employer	1.7 (0.7-3.2)	0.6	2.1	2.5	0.438
Distress financing					
Distress financing (borrowed or sold assets)	28.5 (24.5-32.8)	29.4	28.8	26.3	0.876
Borrowed money for treatment	26.5 (22.6-30.6)	26.9	26.7	25.0	0.948
Sold assets or property	2.5 (1.3-4.3)	2.5	2.1	3.8	0.710
Formal loans (bank or cooperative)	8.8 (6.4-11.6)	13.1	7.9	2.5	0.019*
Online crowdfunding	0.2 (0.0-1.2)	0.0	0.4	0.0	-
Coping strategy score and diversity					
Mean strategies used (SD), range: 0-11	1.99 (0.92)	1.95 (0.85)	1.97 (0.96)	2.13 (0.96)	-
1 strategy only	34.0	-	-	-	-
2 strategies	41.0	-	-	-	-
3 or more strategies	25.0	-	-	-	-
Formal protection effectiveness					
Insurance alleviated burden (among insured, n=68)	54.4	-	-	-	-
Pension reduced impact (among pensioners, n=50)	30.0	-	-	-	-

Table 5 shows the distribution of distress-financing components across financial-exposure subgroups. Distress financing concentrated among those with catastrophic expenditure (36.1% vs 12.4% non-CHE; p<0.001) and impoverishment (31.3% vs 14.8%; p=0.003). Borrowing carried the strongest associations (33.6% vs 11.1%, p<0.001 by CHE; 29.1% vs 13.6%, p<0.01 by impoverishment), while asset sale was extremely rare except among the financially exposed (4.0% CHE vs 0.0% non-CHE; p<0.01). Insurance status showed no protective association - distress-financing rates were virtually identical between insured (27.9%) and uninsured (28.6%) patients (p=0.906). Among insured patients, only 54.4% reported that insurance had alleviated their financial burden; among pensioners, only 30.0% perceived a meaningful reduction in financial impact.

**Table 5:** Distress financing components among chronic NCD patients by catastrophic expenditure, impoverishment, multimorbidity, and insurance status Chi-square test between subgroup pairs. CHE = OOPE ≥10% of household income. Impov. = post-OOPE per capita income below the national poverty line. MM = multimorbidity (≥2 concurrent chronic conditions). * p<0.05, ** p<0.01, *** p<0.001.

Coping behaviour	CHE Yes %	CHE No %	Impov. Yes %	Impov. No %	MM Yes %	MM No %	Insured %	Uninsured %
Distress financing (borrowed or sold)	36.1***	12.4***	31.3**	14.8**	32.0	27.4	27.9	28.6
Borrowed money for treatment	33.6***	11.1***	29.1**	13.6**	28.7	25.7	25.0	26.7
Sold assets for treatment	4.0**	0.0**	3.3*	0.0*	4.1	1.7	2.9	2.4

Predictors of foregone care and distress financing

Table 6 presents the unadjusted and adjusted predictors of foregone care (Model A) and distress financing (Model B) on the full sample (n=480). Table 7 provides the corresponding average marginal effects and adjusted predicted probabilities. Financial-exposure intensity emerged as an independent predictor of both outcomes. Log-transformed OOPE predicted foregone care (AOR: 1.17, 95% CI: 1.00-1.36, p=0.049; AME +3.8 percentage points per unit of log OOPE) and distress financing (AOR: 1.24, 95% CI: 1.04-1.48, p=0.015; AME +4.2 pp per unit). The adjusted predicted probability of distress financing rose from 19.4% at low OOPE (log≈8.0) to 38.1% at high OOPE (log≈12.5) - an 18.7 percentage point gradient driven by exposure intensity alone. After adjusting for sociodemographic characteristics, no demographic variable independently predicted whether a patient rationed care. Facility tier was independently associated with foregone care: secondary (AOR: 0.63, p=0.031; AME -11.5 pp) and tertiary (AOR: 0.54, p=0.048; AME -15.2 pp) patients faced significantly lower odds of care barriers than primary patients, with adjusted predicted probabilities of 53.2% at primary, 41.9% at secondary, and 38.2% at tertiary. For distress financing, pension status conferred a significant protective effect (AOR: 0.40, 95% CI: 0.18-0.89, p=0.025; AME -15.2 pp): pension holders had an adjusted probability of 15.0% compared to 30.2% among those without pension income - a 15.2 percentage point difference. Household size was independently associated with lower odds of distress financing (AOR: 0.89, 95% CI: 0.79-0.99, p=0.040; AME -2.3 pp per additional person), consistent with larger households distributing the burden of chronic illness costs across more income earners and reducing the probability that any single shock triggers distress-level coping. Both models showed good calibration (Hosmer-Lemeshow p=0.412 and p=0.406) and modest discrimination (AUROC: 0.575 and 0.623), with adequate events-per-variable ratios (54.0 and 19.6). The modest AUROC values indicate substantial residual variance not captured by the measured predictors, and the regression coefficients should therefore be interpreted as identifying independently associated exposures rather than as complete explanations of who forgoes care or resorts to distress financing.

**Table 6:** Unadjusted and adjusted logistic regression for predictors of foregone care (Model A) and distress financing (Model B) Parsimonious models confirmed by likelihood-ratio test against full nine-predictor models. Model A retains log OOPE and facility tier; Model B retains sex, pension status, household size, log OOPE, and treatment duration. Treatment duration was retained as a block (joint Wald χ²(3)=6.37, p=0.095; individual categories: 1-4 years p=0.015, 5-9 years p=0.057), consistent with the p<0.10 retention threshold. UOR = unadjusted odds ratio; AOR = adjusted odds ratio; EPV = events per estimated coefficient. * p<0.05.

Predictor	Model A UOR	Model A AOR (95% CI)	Model A p	Model B UOR	Model B AOR (95% CI)	Model B p
Financial exposure						
Log total OOPE (per unit)	1.08*	1.17 (1.00-1.36)*	0.049*	1.15*	1.24 (1.04-1.48)*	0.015*
Treatment duration						
<1 year (ref)	1.00	-	-	1.00	-	-
1-4 years	-	-	-	1.60	2.07 (1.15-3.73)*	0.015*
5-9 years	-	-	-	1.43	1.82 (0.98-3.39)	0.057
≥10 years	-	-	-	1.16	1.49 (0.79-2.83)	0.219
Pension status						
No pension (ref)	1.00	-	-	1.00	-	-
Receiving pension income	1.14	-	-	0.44*	0.40 (0.18-0.89)*	0.025*
Household size						
Per additional person	1.03	-	-	0.93	0.89 (0.79-0.99)*	0.040*
Sex						
Female (ref)	1.00	-	-	1.00	-	-
Male	1.07	-	-	1.26	1.39 (0.91-2.11)	0.124
Facility tier						
Primary (ref)	1.00	-	-	1.00	-	-
Secondary	0.71*	0.63 (0.41-0.96)*	0.031*	0.97	-	-
Tertiary	0.72*	0.54 (0.29-0.99)*	0.048*	0.86	-	-
Model performance						
n	480	480	-	480	480	-
Events	216	216	-	137	137	-
Events per variable (EPV)	54.0	54.0	-	27.4	19.6	-
AUROC	-	0.575	-	-	0.623	-
Hosmer-Lemeshow p	-	0.412	-	-	0.406	-
LR test vs full model p	-	0.325	-	-	0.638	-
Joint Wald test (treat_dur) p	-	-	-	-	0.095	-

**Table 7:** Average marginal effects and adjusted predicted probabilities from parsimonious logistic regression models AME = average marginal effect; APP = adjusted predicted probability; pp = percentage points. AMEs computed at observed covariate values. APPs by subgroup represent average predicted probabilities across all observed covariate combinations. OOPE-level APPs set log OOPE to the specified level with all other covariates at observed values. * p<0.05, ** p<0.01.

Predictor or subgroup	AME or APP	95% CI	p
Model A - Average marginal effects on probability of foregone care			
Log total OOPE (per unit increase)	+3.8 pp*	0.1-7.5 pp	0.046*
Secondary vs primary facility	−11.5 pp*	−21.8 to −1.1 pp	0.030*
Tertiary vs primary facility	−15.2 pp*	−29.9 to −0.5 pp	0.043*
Model A - Adjusted predicted probability by facility tier			
Primary care facilities	53.2%	45.2-61.3%	-
Secondary care facilities	41.9%	35.7-48.1%	-
Tertiary care facilities	38.2%	27.1-49.3%	-
Model B - Average marginal effects on probability of distress financing			
Log total OOPE (per unit increase)	+4.2 pp*	0.9-7.6 pp	0.013*
1-4 years vs <1 year treatment	+13.7 pp*	3.1-24.4 pp	0.011*
5-9 years vs <1 year treatment	+11.0 pp	−0.1 to 22.2 pp	0.053
≥10 years vs <1 year treatment	+7.0 pp	−4.1 to 18.2 pp	0.216
Pension (yes vs no)	−15.2 pp**	−25.8 to −4.6 pp	0.005**
Household size (per person)	−2.3 pp*	−4.5 to −0.1 pp	0.037*
Male vs female sex	+6.4 pp	−1.7 to 14.6 pp	0.121
Model B - Adjusted predicted probability by treatment duration			
<1 year	20.4%	13.2-27.6%	-
1-4 years	34.1%	26.4-41.9%	-
5-9 years	31.4%	23.0-39.9%	-
≥10 years	27.4%	19.1-35.8%	-
Model B - Adjusted predicted probability by pension status			
No pension income	30.2%	25.9-34.5%	-
Receiving pension income	15.0%*	5.3-24.6%	0.005**
Model B - Adjusted predicted probability across financial-exposure levels						
Low OOPE (log=8.0, ≈NGN 3,000)	19.4%	12.2-26.7%	-
Moderate-low (log=9.5, ≈NGN 13,360)	24.8%	20.0-29.6%	-
Moderate-high (log=11.0, ≈NGN 59,900)	31.1%	26.5-35.7%	-
High OOPE (log=12.5, ≈NGN 268,300)	38.1%	29.0-47.3%	-

Treatment duration independently predicted distress financing: patients treated for one to four years faced more than twice the odds of distress financing compared to those treated for less than one year (AOR: 2.07, 95% CI: 1.15-3.73, p=0.015; AME +13.7 pp), and those treated for five to nine years showed a borderline-significant elevation (AOR: 1.82, 95% CI: 0.98-3.39, p=0.057; AME +11.0 pp). The non-monotonic pattern - with the strongest effect at one to four years and attenuation at ≥10 years (AOR: 1.49, p=0.219) - suggests an active financial-burden window during the early-to-mid treatment trajectory, after which households may have exhausted distress-financing options or adapted through alternative coping strategies. The adjusted predicted probability of distress financing was 20.4% among patients treated for less than one year, rising to 34.1% at one to four years before declining to 27.4% at ≥10 years.

The full nine-predictor model results are reported alongside parsimonious models in Appendix 1 to permit assessment of variable selection.

## Discussion

This study identifies three converging mechanisms through which chronic NCD care in Lagos public facilities becomes a financial crisis: pervasive affordability barriers, hierarchical and largely informal coping that culminates in distress financing for a substantial minority, and the independent role of financial-exposure intensity, alongside sociodemographic and structural factors, in shaping who ultimately bears hardship.

Affordability barriers proved pervasive across the cohort. Forty-five percent of patients had been unable to access care because they could not pay, 41.9% identified cost as the primary driver of treatment decisions, and 42.9% had been offered treatments their households could not absorb. Within this aggregate picture, medication unaffordability stands out as the dominant component (57.3% of those offered unaffordable treatment) and, most consequentially, is uniformly distributed across primary, secondary, and tertiary facilities (p=0.621). This pattern implicates pharmaceutical pricing and supply-chain failures as system-wide structural problems rather than tier-specific access gaps, aligning with West African scoping evidence on chronic-disease primary care [10] and Nigerian work positioning medication costs as the dominant driver of NCD catastrophic expenditure [5]. A potential counter-argument is that subsidized disease-specific programs - such as for HIV, TB, and malaria - show different point-of-service patterns and could in principle be scaled to chronic NCDs. However, evidence from Lagos suggests that even nominally free programs do not eliminate hardship: in PLHIV cohorts where antiretroviral therapy is provided without charge, 60% of patients still experienced catastrophic expenditure and 30% had borrowed money to pay for medical care [28]. Medication subsidy at the point of service is necessary but not sufficient; ancillary costs continue to drive financial hardship even when the direct drug cost is removed. The tier gradients we observe in cost-driven decisions (50.0% primary vs 32.5% tertiary; p=0.021) and clinic non-attendance (35.6% vs 10.0%; p<0.001) further underline that primary care - where the least financially capable patients seek care - bears the most concentrated rationing pressure.

The coping patterns we identified mirror the McIntyre-Cleary framework's predicted progression from self-directed resources, through social-network mobilization, to distress financing [6,11], and reproduce the broader sub-Saharan African pattern of informal reliance on cash, asset sale, and borrowing among populations excluded from insurance [14]. The tier-stratified detail tells a more granular story than the composite averages alone: tertiary patients drew most heavily on contributions from relatives (56.3% vs 35.6% at primary; p=0.003), while secondary patients leaned most on children (46.7% vs 30.6% at primary; p=0.002), and three-quarters of the cohort mobilized two or more strategies simultaneously. These gradients suggest that as absolute cost climbs, households mobilize across an expanding circle of intergenerational and extended-family resources- a pattern consistent with household-coping literature on chronic illness in LMIC settings [11]. The tier gradient in formal-loan use (13.1% primary vs 2.5% tertiary; p=0.019) reinforces this reading: primary-tier patients, with the thinnest savings cushion, turn most readily to institutional credit when household resources prove insufficient. The 28.5% distress financing prevalence we observe sits notably below South Asian comparators (58% in Bangladesh hospitalization [12]; 72.4% in Indian breast-cancer care [13]). One possibility is stronger social-network buffering in Nigerian settings - 69.6% of patients mobilized informal support, which may delay or substitute for distress financing. A second is measurement asymmetry: South Asian studies typically capture hospitalization episodes where acute care drives bills sharply upward, whereas this outpatient sample reflects the cumulative recurring expense of chronic care management. A third, less reassuring, is under-reporting of borrowing in face-to-face interviews where stigma attaches to debt. Even at 28.5%, distress financing affects nearly three in ten chronic NCD patients in Lagos public facilities - a figure that likely represents the floor of the burden, not its ceiling.

Financial-exposure intensity emerged as the only variable independently associated with both foregone care and distress financing, while no demographic factor - once exposure was accounted for - independently predicted who rationed care. This challenges the dominant CHE-paradigm framing in which population-level predictors (poverty, absent insurance, rural residence) drive hardship [16]; at the point of care, what matters is not who the patient is but how much exposure they are absorbing. Health insurance offered no protective effect against distress financing in the adjusted models (30.1% vs 27.5%; p=0.677). A critical caveat tempers this finding: only 14.2% of the cohort held any form of insurance (n=68), and an even smaller 3.8% (n=18) actually used their insurance to pay for the current illness. Even when patients held coverage, very few drew on it for chronic NCD care - strong evidence that current Nigerian benefit packages do not cover the recurrent medication and diagnostic costs driving chronic NCD expenditure [5,18]. The null protective effect could therefore reflect limited statistical power to detect a real but modest effect, selection into insurance among patients with already-elevated baseline exposure, or - more substantively - insurance simply not activating at the point of need because the relevant cost components sit outside the benefit envelope. The complementary finding that only 54.4% of insured patients reported meaningful financial-burden alleviation is consistent with each interpretation. Read through Andersen's behavioral model [17], insurance - the most prominent enabling resource for chronic-illness financing - emerges as the protective category most strikingly under-performing in this cohort, while pension income, also classified as enabling but operating through a distinct income-stabilization pathway, retained its protective effect. The pension finding (AOR: 0.40; 15.2 pp protective effect) is statistically robust despite the small holder subgroup (n=50). The interpretation is structurally distinct: pension income functions as a predictable monthly buffer against which recurrent expenses can be absorbed, unconditioned on specific service categories. The counter-argument here is selection - pension holders may differ from non-holders on unobserved characteristics (formal-sector employment history, accumulated savings, household connections) - but the policy implication holds whether the pension effect operates directly through cash flow or indirectly through correlated household characteristics.

Together, these findings reposition what universal health coverage means for chronic NCD patients in Nigeria. Where the standard catastrophic-expenditure paradigm asks how many households cross a threshold, our findings ask, more sharply, which protective mechanisms reduce the probability of crossing it. The answer is multi-component and sequenced: scaling insurance enrolment (in the general population, especially among chronic NCD subgroups) - which operates as the proximal lever that reduces aggregate OOPE; benefit-package redesign that absorbs the recurrent cost driver, pharmaceutical pricing that reduces the cost driver at source, and income protection that stabilizes households against the cost driver they cannot escape function as the complementary distal levers. None of these levers alone is sufficient, and each addresses a distinct mechanism through which the financial burden of chronic illness is produced and sustained.

Policy relevance

These findings carry direct implications for Nigeria's universal health coverage trajectory and for the wider set of LMIC contexts, where chronic NCD care is paid for predominantly out of pocket and insurance coverage remains thin. The proximal policy lever - and the one most immediately actionable under the 2022 NHIA Act - is scaling insurance enrolment among chronic NCD subgroups, given the very low 14.2% baseline coverage in our cohort. Targeted enrolment campaigns through the National Health Insurance Scheme (NHIS) and state-level social health insurance schemes, focused on chronic NCD patient subgroups, would reduce aggregate OOPE and the catastrophic expenditure it generates. The distal - and equally necessary - lever is benefit-package redesign: NHIS packages and the state-level schemes operating under the 2022 Act must absorb recurrent medication, diagnostic monitoring, and tier-appropriate specialist consultations for the most prevalent chronic NCDs, so that expanded enrolment translates into protection at the point of care. The same proximal-then-distal logic generalizes across LMIC settings, including Bangladesh [12], India [13], Tanzania [14], and other sub-Saharan African settings, where chronic illness care remains inadequately protected financially, leaving households exposed to substantial out-of-pocket spending, catastrophic expenditure, and distress financing.

Furthermore, pharmaceutical pricing and essential medicine reforms (including stronger public procurement, generic availability, and supply-chain reliability) address cost drivers that insurance reform alone cannot resolve [29]. Income-protection mechanisms modelled on the Vulnerable Group Fund architecture within the NHIA Act [18] and on conditional cash-transfer schemes operating across LMICs [30] would embed the income-stabilization pathway alongside coverage expansion. The combined package, rather than any single instrument, is what the protective patterns in this cohort suggest will reduce chronic-illness hardship at scale.

Study limitations

Several limitations warrant consideration in this study. The cross-sectional design precludes causal inference; concurrent measurement of expenditure and coping responses limits inference about temporal ordering, and the cumulative evolution of coping strategies over the illness trajectory cannot be traced. Self-reported outcomes may be affected by recall and social-desirability bias, particularly for distress financing and cost-related forgone care, which are prone to under-reporting; even though we tried to mitigate this by cross-checking expenditure figures against receipts, electronic payment statements, pharmacy records, and clinical or facility records where available, not all respondents had complete documentation; therefore, some expenditure estimates may be subject to recall inaccuracy. Although participants were systematically recruited within facilities, the facilities were purposively selected to ensure tier representation. This limits the generalizability of tier-specific findings, particularly because the tertiary sample came from a single institution (n=80) and contributes to several subgroup contrasts; therefore, tertiary-tier estimates should be interpreted cautiously. Facility-based sampling may also underestimate the true population burden by excluding individuals unable to seek formal care. The fieldwork of this study occurred between December 2024 and February 2025, during a period of continued exchange-rate uncertainty following substantial naira depreciation in 2024; therefore, the real-value interpretation of OOPE and poverty-line conversions may be sensitive to exchange-rate assumptions. Finally, modest model discrimination, with AUROC values of 0.575 and 0.623, suggests residual variation from unmeasured factors such as clinical severity, medication burden, household assets, and social-support networks. Longitudinal and community-based studies are needed to clarify causal pathways and estimate the full burden of chronic NCD-related financial hardship in this setting.

## Conclusions

Chronic NCD care in Lagos public facilities becomes a financial crisis through three converging mechanisms: pervasive affordability barriers across all tiers, hierarchical and largely informal coping that culminates in borrowing or asset sale for nearly three in ten patients, and financial-exposure intensity and treatment chronicity that together drive care rationing and distress financing, with the probability of distress financing peaking during the active financial-burden window of one to four years of treatment. Nigeria's path to genuine financial protection for chronic NCD patients runs through a sequenced policy package, including scaling insurance enrolment among chronic NCD subgroups as the proximal lever to reduce OOPE, with benefit-package redesign that absorbs recurrent medication costs, pharmaceutical-pricing reform that targets the system-wide cost driver, and income-protection mechanisms operating as the complementary distal levers that stabilize households against the recurring rhythm of chronic illness expense.

## Appendices

Appendix 1

**Table 8:** Logistic regression results: full nine-predictor models versus parsimonious models for care barriers due to cost (Model A) and distress financing (Model B) Note: Values are odds ratios with 95% confidence intervals in brackets. Model A outcome: foregone care due to cost. Model B outcome: use of distress financing strategies. Full models adjust for all nine predictors; parsimonious models retain only predictors significant at p<0.10 in the full model, confirmed by likelihood-ratio test. Blank cells indicate the predictor was not retained in the parsimonious model for that outcome. * p < 0.05, ** p < 0.01, *** p < 0.001.

Predictor	Model A: Full	Model A: Parsimonious	Model B: Full	Model B: Parsimonious
Age				
Age in years	1.00 (0.99, 1.02)		0.99 (0.97, 1.01)	
Sex				
Sex (male = 1)	1.25 (0.84, 1.86)		1.53 (0.99, 2.39)	1.39 (0.91, 2.11)
Education				
No formal education (ref)	1.00 (ref.)		1.00 (ref.)	
Primary education	1.18 (0.46, 3.02)		0.73 (0.26, 2.03)	
Secondary education	1.03 (0.43, 2.49)		0.79 (0.30, 2.05)	
Tertiary education	0.62 (0.25, 1.55)		0.54 (0.20, 1.46)	
Employment				
Employed	1.13 (0.72, 1.77)		1.17 (0.71, 1.94)	
Health insurance				
Has health insurance	0.85 (0.48, 1.51)		1.31 (0.70, 2.45)	
Pension status				
No pension (ref)	1.00 (ref. )		1.00 (ref.)	1.00 (ref.)
Currently receives pension	1.31 (0.65, 2.62)		0.49 (0.20, 1.19)	0.40* (0.18, 0.89)
Household size				
Household size, persons	0.99 (0.90, 1.10)		0.89 (0.79, 1.00)	0.89* (0.79, 0.99)
Multimorbidity				
Multimorbidity (≥2 conditions)	1.20 (0.77, 1.87)		0.99 (0.60, 1.63)	
Treatment duration				
<1 year (ref)	1.00 (ref.)		1.00 (ref.)	1.00 (ref.)
1–4 years	1.44 (0.85, 2.46)		2.17* (1.19, 3.97)	2.07* (1.15, 3.73)
5–9 years	1.28 (0.73, 2.26)		2.00* (1.05, 3.79)	1.82 (0.98, 3.39)
≥10 years	1.41 (0.79, 2.51)		1.57 (0.81, 3.06)	1.49 (0.79, 2.83)
Facility tier				
Primary facility (ref)	1.00 (ref.)	1.00 (ref.)	1.00 (ref.)	
Secondary facility	0.59* (0.38, 0.91)	0.63* (0.41, 0.96)	0.75 (0.47, 1.21)	
Tertiary facility	0.54 (0.29, 1.02)	0.54* (0.29, 0.99)	0.55 (0.27, 1.13)	
Financial exposure				
Log total out-of-pocket expenditure	1.20* (1.01, 1.42)	1.17* (1.00, 1.36)	1.35** (1.10, 1.66)	1.24* (1.04, 1.48)
Model performance				
Observations	480	480	480	480
Pseudo R²	0.031	0.011	0.046	0.034
LR χ²	20.78	7.26	26.56	19.56
Prob > χ²	0.187	0.064	0.047	0.007

When Paying for Care Becomes a Crisis: Affordability Barriers, Coping Strategies, and Financial Consequences of Chronic Illness in Lagos, Nigeria Study Questionnaire

Serial Number: ________________

This questionnaire is part of a facility-based study investigating affordability barriers, financial coping strategies, and the financial consequences of chronic non-communicable disease (NCD) care among adult outpatients in Lagos State public health facilities. Your responses will help identify where the cost of care becomes unaffordable and how households cope when it does. Participation is voluntary; you have been selected through a systematic random procedure and may decline to answer any question or withdraw at any time without affecting the care you receive. All information will be kept strictly confidential, used solely for research purposes, and reported only in de-identified, aggregate form. Before providing any response here, you must have received a consent form which you are expected to read carefully, ask questions where needed, and then sign. This instrument is interviewer-administered and provided in English, Yoruba, Igbo, and Nigerian Pidgin per participant preference. Kindly answer as accurately as possible by circling the number that corresponds to your answer, or ticking (✓) all options that apply for multiple-response questions.

Ethical approval: Research and Ethics Committee, General Hospital Ikorodu (Reference: GHIKD/REC/35522). Written informed consent was obtained prior to enrolment.

FOR INTERVIEWER / FACILITY USE ONLY

Facility name: _____________________

Facility tier:  1 = Primary   2 = Secondary   3 = Tertiary

Local Government Area: _____________________   Senatorial district: _____________________

Date of interview (dd/mm/yyyy): ____ / ____ / ______   Interviewer ID: ________

Section A: Sociodemographic and Household Characteristics

Predisposing and enabling factors (Andersen Behavioral Model)

A1. Age ________________________  (completed years)

A2. Sex

1 = Male   2 = Female   3 = I would rather not disclose

A3. What is your highest level of education completed?

1 = No formal education   2 = Primary   3 = Secondary   4 = Tertiary

A4. What is your current employment status?

1 = Employed   2 = Self-employed   3 = Unemployed   4 = Not economically active (e.g., retired, homemaker, student)

A5. Occupation (if employed or self-employed) ________________________

A6. What is your current marital status?

1 = Single (never married)   2 = Married   3 = Separated or divorced   4 = Widowed

A7. Total number of people in your household (including yourself) ________________________

A8. Number of children in the household under 18 years ________________________

A9. Number of adults in the household aged 65 years or older ________________________

Section B: Chronic Illness Diagnosis and Treatment Duration

Need factors (Andersen Behavioral Model)

B1. Have you been receiving ongoing treatment for a chronic (long-standing) illness for three months or more?

1 = Yes   2 = No  (if No, participant is not eligible - end interview)

B2. Which of the following chronic conditions have you been receiving treatment for? (Tick all that apply)

   a) High blood pressure / hypertension

   b) Heart problems / cardiovascular disease

   c) Diabetes / high blood sugar

   d) Cancer (specify type: ____________________)

   e) Sickle cell disease

   f) Bone or joint disease

   g) Respiratory disease (e.g., asthma, COPD)

   h) Chronic kidney disease

   i) Chronic liver disease

   j) Mental health condition

   k) Thyroid disease

   l) Other (specify: ____________________)

Note: Multimorbidity is derived as two or more conditions ticked above (≥2 = multimorbid).

B3. How long have you been receiving treatment for this condition? ________________________  (months / years - specify)

Section C: Household Economic Status and Out-of-Pocket Health Expenditure

Financial-exposure intensity

C1. Are you currently working (in any capacity, paid or self-employed)?

1 = Yes   2 = No

Please provide your best estimate for each item below, in Naira per month.

#Item                                                                                                NGN / month

C2

Household income before your illness began

C3

Your personal income before your illness began

C4

Household income since your illness began

C5

Your personal income since your illness began

C6

Total household monthly expenditure (all items)

C7

Total household monthly health expenditure (all members)

C8a

  - of which: medications / drugs

C8b

  - of which: laboratory / diagnostic investigations

C8c

  - of which: hospital / consultation fees

C8d

  - of which: hospital admission costs

C8e

  - of which: other treatment costs

C8f

  - of which: transport to and from the facility

C9

Household monthly non-health expenditure

C10

Household monthly food expenditure

Section D: Health Insurance and Pension Status

Enabling resources (Andersen Behavioral Model)

D1. How do you usually pay for your healthcare?

1 = Out of pocket, always   2 = Private health insurance   3 = Employer/company health insurance   4 = Government health insurance (e.g., NHIS, ILERA EKO)   5 = A combination (specify: ____________________)

D2. Do you currently have any form of health insurance?

1 = Yes   2 = No

D3. If insured, how much do you contribute monthly? ________________________  (NGN)

D4. Have you used your health insurance to pay for care for this illness?

1 = Yes   2 = No   3 = Not applicable (uninsured)

D5. (If insured) Has your health insurance meaningfully reduced the financial impact of this illness on your household?

1 = Yes   2 = No

D6. Do you currently receive any pension income?

1 = Yes   2 = No

D7. (If receiving pension) Has your pension income meaningfully reduced the financial impact of this illness on your household?

1 = Yes   2 = No

Section E: Affordability Barriers to Healthcare

Affordability defined as the fit between the cost of care and household capacity to pay

Framework note: Items in this section operationalize the Cleary et al. (2013) affordability framework, in which affordability barriers manifest as delayed, modified, or abandoned care when cost exceeds a household's capacity to pay.

E1. Are you currently able to pay for the medication needed for your chronic illness?

1 = Yes   2 = No

E2. Are you currently able to pay for the investigations (laboratory / diagnostic tests) needed for your chronic illness?

1 = Yes   2 = No

E3. Are you currently able to pay for hospital admission, where this has been necessary?

1 = Yes   2 = No

E4. Have you ever been offered a treatment for your chronic illness that you were not able to afford?

1 = Yes   2 = No

If Yes, which type(s)? (Tick all that apply)

   a) Medications or drugs

   b) Investigations or diagnostic tests

   c) Surgery

   d) Physiotherapy

   e) Radiotherapy

   f) Other (specify: ____________________)

E5. Do you attend clinic appointments regularly, as scheduled?

1 = Yes   2 = No

If No, what is the main reason? (Select one)

1 = Lack of money   2 = Long distance to facility   3 = Forgetfulness   4 = Industrial action / strikes   5 = Other (specify: ____________________)

E6. What mainly guides your treatment decisions?

1 = Cost / money available   2 = Doctor's clinical recommendation   3 = Family decision

E7. Have there been times when you were unable to obtain medical care simply because you could not afford it?

1 = Almost always   2 = Often   3 = Sometimes   4 = Seldom   5 = Never   6 = Pays extra and manages

Section F: Financial Coping Strategies

Items in this section follow the McIntyre et al. [11] household financial-coping hierarchy, which describes progression from self-directed and formal resources, through social-network mobilization, to distress financing (borrowing or asset sale) when other sources are exhausted.

F1. Self-directed and formal coping

In paying for care for this illness, have you drawn on any of the following? (Tick all that apply)

   i) Personal or household income / savings

   ii) Health insurance

F2. Social-network coping

Have you received any of the following forms of support to pay for care? (Tick all that apply)

   iii) Contributions from relatives

   iv) Contributions from children

   v) Contributions from friends

   vi) Contributions from employer or co-workers

   vii) Contributions from church

   viii) Contributions from mosque

   ix) Online crowdfunding

F3. Distress financing

F3a. Have you ever had to borrow money for any aspect of your treatment?

1 = Yes   2 = No

If Yes, from which source(s) did you borrow? Tick all that apply.

a) Cooperative society

b) Bank

c) Church/religious organization

d) Friends

e) Family members/relatives

f) Employer/co-workers

g) Money lender

h) Other, specify: _________

F3b. Have you ever had to sell household assets or property to pay for this illness?

1 = Yes   2 = No

THANK YOU FOR YOUR TIME AND PARTICIPATION
